# Supplementary material for: Evidence supported by Mendelian randomization: impact on inflammatory factors in knee osteoarthritis
Source: Front Med (Lausanne). 2024 May 28;11:1382836. doi: 10.3389/fmed.2024.1382836 (PMC11165061; doi:10.3389/fmed.2024.1382836)
Supplement: Supplementary file 1 [file Data_Sheet_1.ZIP › data/GCST90274847_5e6/847.forest.pdf]

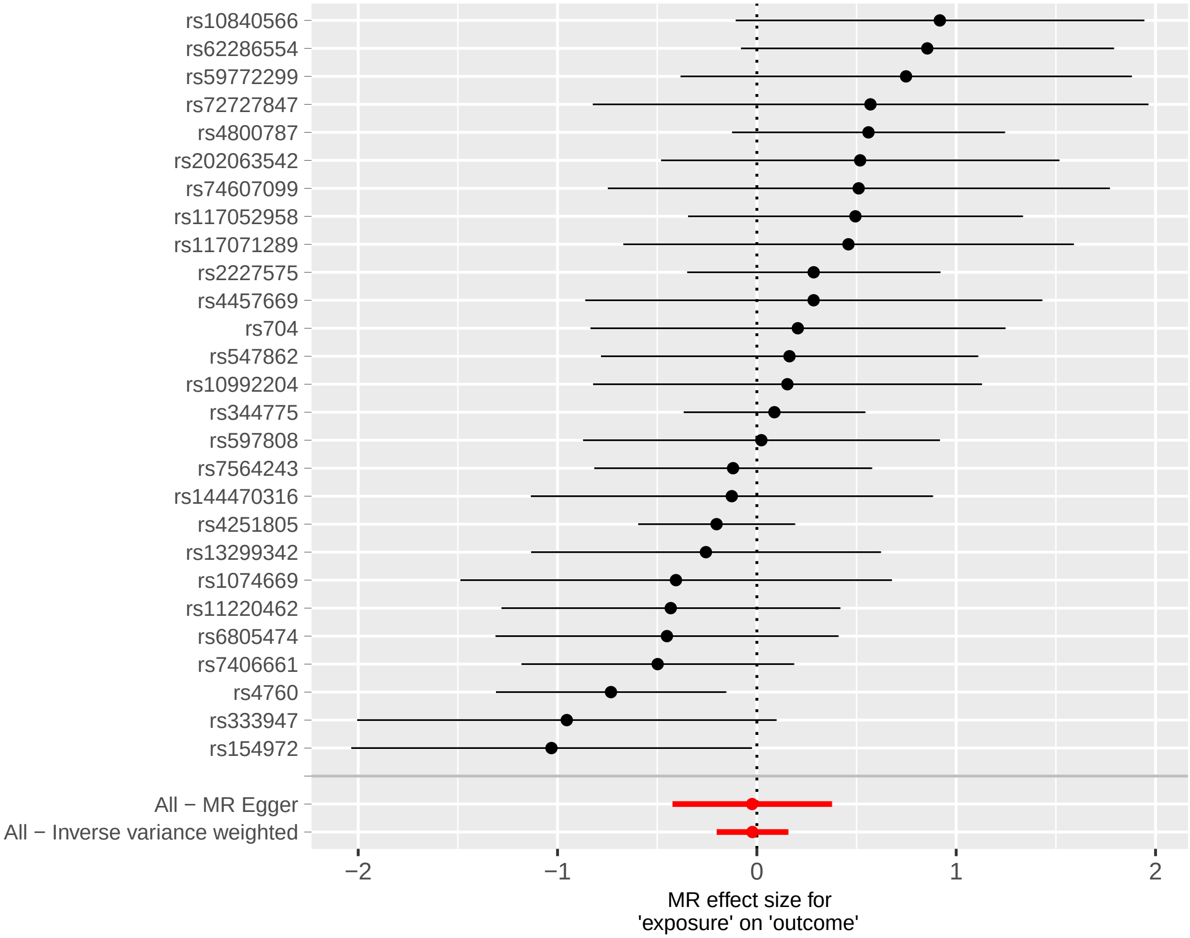
rs10840566
rs62286554
rs59772299
rs72727847
rs4800787
rs202063542
rs74607099
rs117052958
rs117071289
rs2227575
rs4457669
rs704
rs547862
rs10992204
rs344775
rs597808
rs7564243
rs144470316
rs4251805
rs13299342
rs1074669
rs11220462
rs6805474
rs7406661
rs4760
rs333947
rs154972
All − MR Egger
All − Inverse variance weighted
−2
−1
0
1
2
MR effect size for
'exposure' on 'outcome'
